# Climate Change, Crop Yields, and Undernutrition: Development of a Model to Quantify the Impact of Climate Scenarios on Child Undernutrition

**DOI:** 10.1289/ehp.1003311

**Published:** 2011-08-15

**Authors:** Simon J. Lloyd, R. Sari Kovats, Zaid Chalabi

**Affiliations:** Department of Social and Environmental Health Research, London School of Hygiene and Tropical Medicine, London, United Kingdom

**Keywords:** cereal crops, climate change, Monte Carlo simulation, quantitative model, undernourishment, undernutrition

## Abstract

Background: Global climate change is anticipated to reduce future cereal yields and threaten food security, thus potentially increasing the risk of undernutrition. The causation of undernutrition is complex, and there is a need to develop models that better quantify the potential impacts of climate change on population health.

Objectives: We developed a model for estimating future undernutrition that accounts for food and nonfood (socioeconomic) causes and can be linked to available regional scenario data. We estimated child stunting attributable to climate change in five regions in South Asia and sub-Saharan Africa (SSA) in 2050.

Methods: We used current national food availability and undernutrition data to parameterize and validate a global model, using a process-driven approach based on estimations of the physiological relationship between a lack of food and stunting. We estimated stunting in 2050 using published modeled national calorie availability under two climate scenarios and a reference scenario (no climate change).

Results: We estimated that climate change will lead to a relative increase in moderate stunting of 1–29% in 2050 compared with a future without climate change. Climate change will have a greater impact on rates of severe stunting, which we estimated will increase by 23% (central SSA) to 62% (South Asia).

Conclusions: Climate change is likely to impair future efforts to reduce child malnutrition in South Asia and SSA, even when economic growth is taken into account. Our model suggests that to reduce and prevent future undernutrition, it is necessary to both increase food access and improve socioeconomic conditions, as well as reduce greenhouse gas emissions.

Hunger and undernutrition are pervasive, thought to be worsening in absolute terms, and are major contributors to global ill health [Black et al. 2008; Food and Agricultural Organization of the United Nations (FAO) 2009]. More than one billion people are undernourished (FAO 2009), and about a third of the burden of disease in children < 5 years of age is attributable to undernutrition (Black et al. 2008). Economic growth is anticipated by many to reduce future undernutrition (Smith and Haddad 2002), although recent observations do not support this assumption (Subramanyam et al. 2011).

Global food security depends on a range of factors (Schmidhuber and Tubiello 2007), with cereal production playing a major role (Parry et al. 2009). Data suggest that global per capita cereal production plateaued during the 1980s and has since declined (Magdoff and Tokar 2010), despite production increases in some regions (FAO 2011). Further, with economic growth, dietary preferences tend toward greater meat consumption, placing greater demands on cereal production to provide animal feed (Msangi and Rosegrant 2011).

Concern is growing that efforts to reduce undernutrition in the coming decades may be threatened by global climate change (Nelson et al. 2010; Parry et al. 2009; Schmidhuber and Tubiello 2007). Scientific assessments indicate that warming will have an overall negative impact on major cereal yields in low-latitude areas, although yields may increase in some high-latitude areas (Easterling et al. 2007). Climate change could place an additional 5–170 million people “at risk of hunger” by the 2080s (Parry et al. 1999, 2004; Rosenzweig and Parry 1994). Food security is now one of the leading concerns associated with anthropogenic climate change (Parry et al. 2009).

A number of terms are used to describe hunger and undernutrition. “Undernourishment” is not a health outcome per se; it is a theoretical model-based estimate of access to calories developed by the FAO and is defined as the proportion of people “whose dietary energy consumption is continuously below a minimum dietary energy requirement for maintaining a healthy life and carrying out light physical activity with an acceptable minimum body-weight for attained-height” (FAO 2010). That is, it has one final cause: a lack of food. “At risk of hunger” is synonymous with undernourishment.

“Undernutrition” refers to a physical state and is measured using (among other things) anthropometric indices such as stunting (height-for-age) and underweight (weight-for-age) [World Health Organization (WHO) 2010]. A lack of food—that is, undernourishment—is one of the many causes of undernutrition, which also include poor water and sanitation provision, low levels of women’s education, repeated episodes of infectious diseases, and low birth weight [United Nations Children’s Fund (UNICEF 1990); for more details on causes, see Black et al. 2008; UNICEF 1990]. Checkley et al. (2008), for example, estimated that 25% [95% confidence interval (CI): 8, 38%] of irreversible stunting at 24 months of age could be attributed to having had five or more episodes of diarrhea. Although it can be argued that undernutrition itself is not a health outcome, undernutrition can be directly linked to increased risk of death and poor health (Black et al. 2008). Additionally, child undernutrition has long-term consequences for the health and earning potential of adults (Victora et al. 2008).

To quantify future health burdens, it is preferable to model undernutrition (which refers to a physical state and accounts for complex causation) rather than undernourishment (which is a theoretical concept). They are often poorly correlated (Klasen 2006; Svedberg 2002) and this suggests that undernourishment is a poor proxy for undernutrition. The WHO concluded that (using a number of simplifying assumptions) undernutrition represented a significant proportion of the total burden of disease estimated to be attributable to climate change in 2000 (McMichael et al. 2004). Only one group has provided more recent quantitative estimates of future undernutrition attributable to climate change. Nelson et al. (2009) reported that, for two climate scenarios, climate change may increase underweight in children < 5 years of age by around 20% by 2050. Underweight was estimated using an equation developed by Smith and Haddad (2000), which is driven by per capita calorie availability and socioeconomic indicators: the ratio of female to male life expectancy, female enrollment in secondary education, and access to improved water supply. Future per capita calorie availability was estimated by modeling crop yield and global food trade. All other nonclimate factors were assumed to stay constant over time (i.e., unchanged from baseline values). These assumptions are likely to have led to an overestimate of the future burden attributable to climate change because this approach assumes that living conditions in countries will improve little over the next 40 years. This is not consistent with historical trends; between 1970 and 1995, 43% of the reduction in child underweight has been attributed to improved female education, compared with 26% for increased food availability and 19% from improved water access (Smith and Haddad 2000).

More recently, the same group produced updated estimates for a broader range of scenarios using a similar strategy (Nelson et al. 2010). Based on expert opinion, the socioeconomic variables driving the underweight model were varied with time but were considered constant across three socioeconomic scenarios broadly representing pessimistic, business-as-usual, and optimistic economic growth.

Despite the importance of socioeconomic influences on health, the data currently available for climate impact studies are largely limited to population and gross domestic product (GDP) projections that were created for estimating future greenhouse gas emission concentrations. At present, any modeling efforts must work within these constraints. However, attention is now being focused on creating a wider range of plausible socioeconomic scenarios for climate impact assessments (Moss et al. 2010).

We developed a parsimonious model for estimating future undernutrition attributable to global climate change, specifically due to its impacts on crop productivity. We then estimated the future impact of climate scenarios on undernutrition in children for five world regions in Africa and Asia in 2050 using previously published estimates of climate change–attributable changes in calorie availability from Nelson et al. (2009). [The more recent estimates (Nelson et al. 2010) are not included in our assessment because they were released after the completion of our project.]

## Materials and Methods

We first describe the development and fitting of a model for estimating the prevalence of stunting. Second, we outline the process of estimating the proportion undernourished (PoU) using per capita calorie availability estimates from Nelson et al. (2009). Finally, we discuss the simulation process for estimating future undernutrition attributable to global climate change.

*Model development.* Our outcome of interest is stunting in children < 5 years of age, because this best captures the impact of conditions over the long term (Black et al. 2008). Children are considered moderately stunted if they are > 2 SDs below the mean expected height-for-age and severely stunted if > 3 SDs below the mean (de Onis and Blossner 2003).

Scenario data are limited essentially to future food availability and per capita GDP, and many causes of stunting cannot be explicitly modeled. We considered stunting to have two main causes, which we refer to as “food causes” and “nonfood causes.” Food causes are represented as PoU, which accounts for climate change effects on calorie availability (via changes in crop productivity) and food access. [Stunting has food causes other than calories, e.g., micronutrient deficiencies (Black et al. 2008), but these are not represented in PoU, nor are they modeled in climate-crop models.] Nonfood causes are represented as a “black box cluster” of socioeconomic factors acting at various levels and represent the wide range of social and demographic causes of stunting, such as low female literacy and poor health care access (Frongillo et al. 1997). Nonfood causes are modeled using per capita GDP and the Gini coefficient for income distribution to generate a “development score,” as described below.

The conceptual model is represented by two general equations:

*y_ijk_* = α*_k_* + β*_k_ x_ij_* + γ*_k_ w_ij_* + θ*_k_ x_ij_w_ij_* [1] for every *i*, *j*; *k* = 2, 3,

*y_ij_*_1_ = 1 – *y_ij_*_2_ – *y_ij_*_3_ [2] for every *i*, *j*; *k* = 1,

where *y_ijk_* is the proportion of children < 5 years of age stunted in country *i*, in region *j*, at level *k*, where *k* is 1 if no/mild stunting, 2 if moderate stunting, or 3 if severe stunting; *x_ij_* is food causes of stunting, represented by the PoU in country *i*, in region *j*; and *w_ij_* is nonfood causes of stunting, represented by the “development score” (defined below) in country *i*, in region *j*. The parameters α*_k_*, β*_k_*, γ*_k_*, and θ*_k_* are to be determined: β*_k_* is the physiological relation between undernourishment and stunting (details given below), γ*_k_* relates the development score to stunting, θ*_k_* relates the interaction between undernourishment and the development score to stunting, and α*_k_* is the regression constant.

Equation 1 is a bilinear model because it is a linear function of the independent variables (*x_ij_* and *w_ij_*) and their product (*x_ij_w_ij_*). After estimating moderate (*y_ij_*_2_) and severe (*y_ij_*_3_) stunting, we estimated the proportion not or mildly stunted (*y_ij_*_1_) as described in Equation 2.

The “development score” is an indicator of the nonfood causes of stunting. It is driven by country-level projections of future per capita GDP and the baseline (i.e., most recent estimate available) Gini coefficient (because no projections were available). The development score is scaled from 0 to 1; it equals 0 when socioeconomic conditions are optimal (in terms of avoiding undernutrition) and all undernutrition is attributable to food causes, and it equals 1 when nonfood causes are at their current (baseline) global maximum [for additional information on development score calculations, see Supplemental Material, Annex 1 (http://dx.doi.org/10.1289/ehp.1003311)].

To parameterize the equations, we assembled a global data set obtaining country-level undernourishment estimates from the FAO (FAO 2010), per capita GDP and Gini data from the World Bank Development Indicators (WBDI) database (World Bank 2010), and stunting data from the WHO’s Global Database on Child Growth and Malnutrition (WHO 2010).

Stunting data were matched to undernourishment data to within a 1-year period. Per capita GDP and Gini coefficient estimates were matched as closely as possible to the stunting data year. The data set covered the period 1988–2008 and contained 186 records with complete data. Countries were included in the data set more than once if they had data for multiple years.

*Fitting the model.* We decided, *a priori*, to use a process-driven (theory-based) rather than a standard data-driven (statistical) approach to develop and parameterize the model equations. The purpose of the model is to describe plausible futures, so we designed it to be driven as much as possible by relationships that will be stable over time.

Of the two model variables, we assumed that food causes have a more stable relationship with stunting than do nonfood causes because food causes are physiologically related to stunting, and it is reasonable to assume that this relationship will hold over the next 50 years. In contrast, we assumed that nonfood causes—which we modeled using per capita GDP and the Gini coefficient—do not necessarily have a stable relationship with stunting because the relationship is mediated, at least partly, by social and political factors that may change over time. Therefore, when fitting our model, we first quantified the relationship between stunting and food causes and then considered socioeconomic factors.

We assumed that if someone had insufficient food, and nonfood causes of stunting were absent (i.e., socioeconomic conditions were optimal in terms of avoiding undernutrition), there would be a predictable risk of stunting; that is, we assumed the relationship between food intake and stunting is physiologically determined and holds globally. This assumption is supported by ample evidence that, at least until 6 years of age, all adequately nourished and optimally cared for children will have similar, predictable growth rates (WHO 2006). In addition to this food intake–related burden, if socioeconomic conditions are poor, there is an additional risk of stunting from nonfood causes and their interaction with food causes, for example, high rates of diarrhea associated with inadequate sanitation. We do not consider it probable that a country will lack sufficient food but otherwise have “optimal” socioeconomic conditions; our conception is theoretical.

Using the data set, we estimated the predictable but unknown physiologically based relationship between undernourishment and stunting at level *k* (β*_k_*) as

β*_k_* = min*_i_*_,_*_j_* {*y_ijk_*/*x_ij_*; *i* = 1…, *j* = 1…}. [3]

(The operator *min_i_*_,_*_j_*{∙} means the minimum of the argument in {∙}.) This minimum proportion was obtained by finding the minimum value of the ratio of *y_ijk_* to *x_ij_* among all the countries in all regions, where, as defined above, *y_ijk_* represents the proportion stunted < 5 years of age in country *i*, in region *j*, and stunting level *k*; and *x_ij_* represents the proportion of the population undernourished in county *i*, in region *j*. Because it is unlikely that all stunting in a country is caused by food causes alone, our estimate of β*_k_* will be an overestimate of the purely physiological relationship between food and stunting. In practice, because the minimum observed value may be too low because of data errors, we chose to use the 5th percentile of the distribution of *y_ijk_*/*x_ij_* as the best estimate of β*_k_* and used the 1st and 10th percentiles as the boundaries of its plausible range (see “Estimating future stunting,” below).

Once the above relationship was found, one-fifth of the data set (37 records) was randomly selected and reserved for model validation; the remainder (149 records) was used to parameterize the equations. (To obtain the best possible estimate, and considering that our method of estimation provides a rough approximation, we used the entire data set to estimate β*_k_*.)

We parameterized the equations in a stepwise manner. In the first step, we used β*_k_* to attribute a proportion of stunting to food causes in all countries in the parameterization data set:

*r_ijk_* = β*_k_ x_ij_* [4] for every *i*, *j*, *k*,

where *r_ijk_* is the proportion of stunting attributable to food causes in country *i*, in region *j*, at level *k*.

In the second step, we attributed the remaining proportion of stunting to nonfood causes and the interaction between food and nonfood causes:

*s_ijk_* = *y_ijk_* – *r_ijk_* [5] for every *i*, *j*, *k*,

where *s_ijk_* is the proportion of stunting attributable to nonfood causes and the interaction between food and nonfood causes in country *i*, in region *j*, at level *k*. We then used linear methods to estimate the parameters (α*_k_*, γ*_k_*, θ*_k_*) of the bilinear model:

*s_ijk_* = α*_k_* + γ*_k_ w_ij_* + θ*_k_ x_ij_w_ij_* [6] for every *i*, *j*, *k.*

The model was validated by comparing levels of stunting predicted by the model to observed stunting in the reserved portion of the data set (37 records).

For α*_k_*, γ*_k_*, and θ*_k_* we used the standard errors of the estimates to describe the plausible range of their true values. We carried out our analysis with Stata (version 11; StataCorp, College Station, TX, USA).

*Estimating future population undernourished.* The model required estimates of future PoU with and without climate change. Calculation of PoU requires data for *a*) the coefficient of variation for within-population calorie distribution, *b*) the average minimum calorie requirements to avoid undernourishment in the population, and *c*) per capita calorie availability (FAO 2003). Because projection data for *a*) and *b*) are not available, we assumed they remain at baseline levels. For *c*), we used estimates made by Nelson et al. (2009) for futures with and without climate change. The future without climate change (reference scenario) was represented with the 1950–2000 climate. The two climate change scenarios were derived from two climate models [the National Centre for Atmospheric Research (NCAR) model and the Commonwealth Scientific and Industrial Research Organisation (CSIRO) model] forced by a medium-high emissions scenario [the Intergovernmental Panel on Climate Change A2 scenario from the Special Report on Emissions Scenarios; see Nakicenovic and Swart (2000)]. The two climate scenarios were used to address uncertainty in the climate system; the NCAR model is warmer and wetter than the CSIRO model. The global average increases in maximum temperature and precipitation over land by 2050 were 1.9°C and 10%, and 1.2°C and 2% for the NCAR and CSIRO scenarios, respectively. For details of the assumptions in the crop modeling (e.g., carbon dioxide fertilization, irrigation, and adaptation responses), extrapolations to other food groups, and the trade model, see Nelson et al. (2009). For additional information on PoU estimation, see Supplemental Material, Annex 2 (http://dx.doi.org/10.1289/ehp.1003311).

*Estimating future stunting.* The principal input to our simulation model was future country-level PoU derived from Nelson et al. (2009). We ensured within-scenario consistency by using the same GDP (G. Nelson, International Food Policy Research Institute, personal communication) and population projections [United Nations medium variant, 2006 revision (United Nations 2007)] used in the calorie availability projections. Our estimates of the Gini coefficient were the most recent estimates available from the WBDI (World Bank 2010).

To account for parameter uncertainty, we used a standard Monte Carlo approach. Each of α*_k_*, γ*_k_*, and θ*_k_* were assumed to be normally distributed about their point estimates as defined by their respective standard errors. β*_k_* was assumed to be uniformly distributed between the 1st and 10th percentiles of the distribution of *y_ijk_*/*x_ij_*. This method produced probability density functions (PDFs) of future stunting.

We aimed to base each PDF on 100,000 estimates. We selected the first 100,000 estimates that were > 0 and < 1. By rejecting low and high estimates, we potentially introduced an upward or downward bias; to assess this, we quantified the proportion of rejected results [see Supplemental Material, Table 1 (http://dx.doi.org/10.1289/ehp.1003311)].

**Table 1:** Summary of the data used to parameterize the model.

No. observations	Children stunted*a* (%)			Undernourished*a *(%)	Per capita GDP*a* (2000 US$)
	Region	Moderate	Severe			Gini*a*^,b^
Global		149		19 (3–30)		16 (1–36)		24 (5–70)		897 (81–5,513)		0.45 (0.17–0.74)
Caribbean		9		8 (3–14)		4 (1–8)		12 (5–27)		2,398 (942–3,688)		0.47 (0.4–0.53)
Central America		12		19 (13–27)		12 (4–29)		19 (5–52)		2,051 (633–5,513)		0.53 (0.49–0.58)
South Asia		8		26 (22–30)		26 (2–35)		22 (16–26)		364 (207–589)		0.38 (0.3–0.47)
Southeast Asia		12		22 (11–27)		18 (3–33)		21 (9–41)		729 (232–1,958)		0.4 (0.33–0.44)
SSA												
Central		5		21 (16–26)		24 (15–35)		49 (21–76)		309 (81–578)		0.51 (0.44–0.61)
East		23		24 (14–29)		23 (12–34)		36 (15–62)		286 (110–757)		0.43 (0.3–0.6)
South		8		30 (19–23)		14 (9–30)		29 (14–46)		1,298 (415–2,599)		0.60 (0.5–0.74)
West		35		20 (13–25)		19 (7–30)		24 (8–51)		315 (138–684)		0.43 (0.36–0.53)
Other regions		37		16 (6–23)		16 (6–23)		18 (5–58)		1,249 (206–3,975)		0.43 (0.17–0.62)
Data are shown globally (for all those countries for which data were available) and for regions defined for the Global Burden of Disease Study (Harvard University et al. 2009). **a**Values are regional means (minimum–maximum); numbers are based on records from between 1991 and 2008. **b**The Gini coefficient ranges from 0, where there is perfect income equality, to 1, where all income goes to one person.												

Final estimates were produced at the regional level for South Asia and four regions in sub-Saharan Africa [SSA; central, east, south, and west; see Supplemental Material, Table 2 (http://dx.doi.org/10.1289/ehp.1003311)]. We aggregated stunting from the country to regional level using population weighting. We ran the simulation using MATLAB (version 2009b; MathWorks, Natick, MA, USA).

**Table 2:** Central estimates and plausible ranges of model parameters.

Level of stunting	β*k*	α*k*	γ*k*	θ*k*
Moderate (*k* = 2)		0.35 (0.20–0.44)		0.025 ± 0.013		0.26 ± 0.028		–0.43 ± 0.041
Severe (*k* = 3)		0.18 (0.11–0.28)		–0.052 ± 0.021		0.34 ± 0.044		–0.18 ± 0.064
β*k* is the physiological relation between undernourishment and stunting [5th percentile (1st–10th percentile)]; α*k* is the regression constant, γ*k* relates the development score to stunting, and θ*k* relates the interaction between undernourishment and the development score to stunting (regression estimate ± SE).								

## Results

*Model development and parameters.* Table 1 summarizes the data set used to parameterize our model. The correlation coefficients between stunting and PoU were 0.16 and 0.19 for moderate and severe stunting, respectively. For univariate analysis of stunting and the development score, *R*^2^ was 0.40 for moderate stunting and 0.45 for severe stunting; when PoU was added to these models, *R*^2^ was unchanged. That is, using a data-driven approach, including PoU as an explanatory variable would not improve the model fit to estimate stunting in the present compared with using the development score alone. This supported our approach using a theory-based model that accounts for both food access and socioeconomic conditions.

The model parameter estimates are shown in Table 2. The β parameter is an estimate of the assumed physiological relationship between a lack of food and stunting. Thus, the central estimate of β = 0.35 for moderate stunting suggests that for every 1% of the population who are undernourished, on average 0.35% of children < 5 years of age will be moderately stunted. Using the validation data set, the predicted and observed values are well correlated, with correlation coefficients of 0.78, 0.66, and 0.80 for no/mild, moderate, and severe stunting, respectively [for scatterplots, see Supplemental Material, Figure 1 (http://dx.doi.org/10.1289/ehp.1003311)].

**Figure 1:**
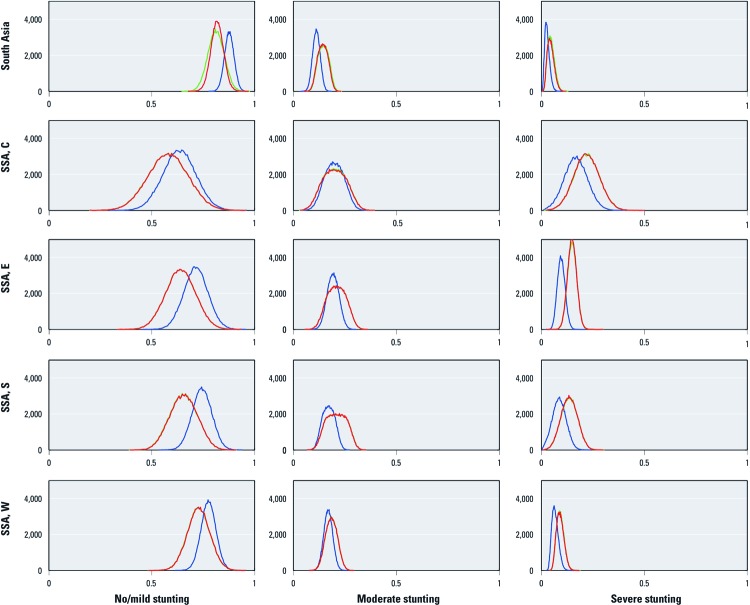
Histograms proportional to the PDFs for the proportion estimated to be stunted in 2050, by region: SSA, C (central); SSA, E (east); SSA, S (south); SSA, W (west).**Histograms were derived from 100,000 Monte Carlo runs. The *x*-axes are proportion stunted at a given level; the *y*-axes are number of estimates. The curves are blue for no climate change, green for NCAR, and red for CSIRO. There is large overlap of the NCAR and CSIRO curves.

*Estimates of future proportions undernourished.* The proportions of regional populations projected to be undernourished in 2050 are shown in Table 3. Countries for which complete data were not available were excluded [see Supplemental Material, Table 2 (http://dx.doi.org/10.1289/ehp.1003311)]. The estimates suggest that climate change will increase PoU compared with a future without climate change, and also that climate change and population growth will increase it to above current levels in all regions.

**Table 3:** Estimates of undernourishment and stunting at baseline (present) and in 2050 with and without climate change (CC).

Percent undernourished*a*							Percent relative increase in PoU under climate change*b*	Percent stunted (mean ± SD) of the PDFs*a,c*									Percent relative increase in stunting under climate change*d*
2050					Stunting level	2050											
Region	Baseline	No CC	NCAR	CSIRO		Baseline		No CC	NCAR	CSIRO							
South Asia		22		15		30		29		97		Moderate		23		11.2 ± 1.8		14.6 ± 2.6		14.3 ± 2.5		29
												Severe		19		2.9 ± 1.2		4.8 ± 1.7		4.6 ± 1.6		61
SSA																						
Central		65		53		81		80		52		Moderate		20		19.9 ± 4.7		20.1 ± 5.7		20.1 ± 5.7		1
												Severe		20		16.8 ± 5.6		22.1 ± 6.1		22.0 ± 6.1		31
East		35		24		52		52		116		Moderate		22		19.3 ± 2.9		21.1 ± 4.6		21.1 ± 4.5		9
												Severe		18		9.7 ± 1.9		15.0 ± 2.3		15.0 ± 2.3		55
South		32		33		60		60		82		Moderate		16		17.1 ± 3.0		21.0 ± 4.8		21.0 ± 4.8		23
												Severe		12		8.8 ± 3.3		13.6 ± 4.0		13.6 ± 4.0		55
West		15		12		29		29		142		Moderate		17		17.0 ± 2.2		18.6 ± 2.9		18.5 ± 2.9		9
												Severe		16		6.8 ± 1.6		9.3 ± 1.8		9.2 ± 1.8		36
**a**Baseline undernourishment and stunting data are from FAO (2010) and are calculated as population-weighted averages using the most recent data available; countries without data are excluded. “No CC” is the reference scenario (i.e. future without climate change); “NCAR” and “CSIRO” are futures under climate change scenarios based on the NCAR and CSIRO models respectively. **b**Compared with a future with no climate change; estimate based on average estimates from NCAR and CSIRO. For example, for South Asia the calculation was: . **c**Empirically derived PDF, derived from the Monte Carlo simulations. **d**Compared with a future with no climate change; estimate based on average of the mean of the estimates from NCAR and CSIRO. For example, for moderate stunting in South Asia the calculation was: .																						

*Projections of stunting in 2050.* We estimate that climate change will increase stunting in all regions (Table 3), with severe stunting increasing by 30–50%. The estimated relative change in stunting was smaller than the estimated relative change in undernourishment. Figure 1 shows the uncertainty in the stunting estimates as histograms of probabilistic outcomes derived from the Monte Carlo simulation.

We compared our stunting estimates with underweight estimates made by Nelson et al. (2009) (Table 4). The results are not directly comparable, but we have assumed that the ratio of underweight to stunting at baseline remains constant in the future. The final column shows this ratio as a regional, population-weighted average calculated using the most recent estimates of underweight and stunting (FAO 2010).

**Table 4:** Model estimates of numbers of children affected by undernutrition in 2050: underweight and stunting.

Millions of children affected by undernutrition in 2050					Additional millions of children affected by undernutrition with climate change			Baseline ratio of underweight to stunting*a*
Region	Outcome	No CC	NCAR	CSIRO	No CC	NCAR		
South Asia		Underweight*b*		52		59		59		7		7		1.1
		Stunting*c*		20		27		26		7		6		
SSA		Underweight*b*		42		52		52		10		10		0.7
		Stunting*c*		45		54		54		9		9		
**a**Calculated as [(moderate + severe underweight)/(moderate + severe stunting)] using data for the present (FAO 2010) and as a regional, population-weighted average. **b**Underweight estimates for 2050 are from Nelson et al. (2009). **c**Stunting estimates are the sum of the numbers moderately and severely stunted, based on the mean estimates of the empirically derived PDFs.														

## Discussion

We have developed the first global model to estimate the impact of climate change on future stunting—a more relevant outcome measure for human population health than “population at risk of hunger” (i.e., undernourishment) or underweight. Additionally, our model distinguishes moderate from severe stunting, which bring substantially different health risks (Black et al. 2008). Based on our conservative assumptions, the model suggests that climate change will have significant effects on future undernutrition, even when the beneficial effects of economic growth are taken into account. This is particularly so for severe stunting, with a 62% increase in South Asia and a 55% increase in east and south SSA. The health implications of this are large: according to Black et al. (2008), moderate stunting increases the risk of all-cause death 1.6 times (95% CI: 1.3, 2.2) and severe stunting increases the risk 4.1 times (95% CI: 2.6, 6.4).

Comparing our results with those of Nelson et al. (2009) should be done cautiously because the outcome measures are different. Our estimates for stunting are lower than estimates from Nelson et al. (2009) for underweight in both South Asia and SSA (Table 4). Our estimates for SSA are closer but still lower. It is likely these differences are largely explained by how the models account for socioeconomic conditions. Nelson et al. (2009) estimated underweight using a complex model that accounted for many socioeconomic factors, but because of a lack of data, all the factors (except for food access) were held at baseline levels. Our stunting equation represents socioeconomics more simply but is able to account for expected changes over the next 40 years. World Bank projections suggest that in South Asia, GDP will increase nine times between 2005 and 2050—an absolute increase of about $7,000 billion (year 2000 US$); in SSA the figures are five times and $1,700 billion. Hence, allowing for these changes results in lower future stunting estimates, with a greater reduction in South Asia.

*Model approximations and assumptions.* We used a theory-based rather than statistically based approach to modeling. Although we accept that a statistical approach would be sound if our aim were to estimate current stunting, our aim was to estimate future stunting. Thus, we developed a model that was driven as much as possible by a relationship that can reasonably be expected to remain constant over time. We assumed that the physiological relationship between stunting and undernourishment will remain constant and approximated this relationship in the first step. After this, because the relationship between stunting and GDP (which is mediated by, among other things, political and social conditions) may vary significantly over time, we fitted the development score and interaction term as a second step.

We made several key approximations in constructing the model. The first approximation was to fit a separate bilinear regression model to two of the stunting levels and then use these to estimate no/mild stunting. Although a more rigorous approach would fit the three regression models simultaneously while ensuring that the proportions (for each country) are positive and always add up to unity, this could lead to an imbalance in the goodness of model fit of one level at the expense of another. The second approximation was to treat the food causes and the product of the food causes and nonfood causes as two independent variables in the least squares fit. This, of course, would introduce errors because the variables are correlated. Nevertheless, the approximation was validated against a data set different from that on which it was based. The third approximation concerns the approach we adopted for the probabilistic (Monte Carlo) simulations. Simulated values that were either < 0 or > 1 were discarded. This could introduce bias, and we quantified this potential. No estimates were rejected for being > 1, meaning there is no risk of downward biasing. For estimates < 0, no moderate stunting estimates were rejected, but severe stunting estimates were rejected in all regions [see Supplemental Material, Table 1 (http://dx.doi.org/10.1289/ehp.1003311)], meaning there is some potential for upward bias. Because more estimates were rejected in the “no climate change” future compared with the “climate change” future, this may have reduced the apparent impact of climate change on severe stunting.

The fourth approximation was the estimate of the physiological relationship between stunting and a lack of food (as represented by undernourishment). We ran our model assuming that a uniform distribution of values between the 1st and 10th percentile of the ratio of stunting to undernourishment adequately represented the true value. In support of our estimates, our parameters suggest that about 60% of stunting could not be directly attributed to a lack of food; this is in line with previous estimates that around 40–60% of undernutrition could be attributed to environmental conditions (predominantly a lack of water and sanitation) (Pruss-Ustun and Corvalan 2006).

Although a more elaborate approach could have been used, inevitably there is always a trade-off between model complexity and ease of model use. We have tilted more toward model simplicity but at the same time quantified the errors induced by the approximations, as far as possible.

We made estimates of future undernourishment from projected calorie availability. In doing so we assumed that both within-country food distribution and average minimum calorie requirement remained at baseline levels. In support of these assumptions, we note that FAO estimates of within-country food distribution are based on extrapolations of infrequently collected data from relatively few countries and are restricted to lie between values representing a given maximum and minimum equity of distribution (based on estimated requirements). Varying values within this range has been found to have little impact on PoU in countries with low calorie availability (FAO 1996; Svedberg 2002). Considering minimum calorie requirements, the estimated mean change in requirements across all countries was just 0.1% per year over the period 1990–1992 to 2004–2006 (FAO 2010). Further, according to FAO data (FAO 2010), the average minimum calorie requirements are increasing in most low-income countries and are higher (and increasing) in middle-income countries. This means our estimate may be conservative. Finally, Svedberg (2002) estimated that over a 20-year period, 88% of the change in regional undernourishment was explained by changes in per capita calorie availability.

We assumed that, once per capita GDP reached $10,000 (2000 US$; with an associated Gini coefficient of 0.38), socioeconomic conditions no longer contributed to stunting. We tested the sensitivity of the model to this assumption by rerunning it without this assumption. This made a negligible difference to estimates (data not shown).

Finally, a limitation of the overall modeling strategy is that climate change is assumed to enter the system only through its impact on crop production. First, this allows only a partial consideration of future food security: food availability and, to a degree, access are modeled, but stability and utilization are not (for a discussion, see Schmidhuber and Tubiello 2007). Second, climate change is likely to affect undernutrition by a variety of routes, including plant diseases, extreme drought events, infectious disease, labor productivity, water availability, and overall impact on GDP. So far, these aspects have not been accounted for, and we recommend that future assessments (of all health impacts, not just undernutrition) attempt to account for the multiple effects of climate change.

*Model behavior.* We examined model behavior over the range of plausible input variable values. When either undernourishment or the development score are high (a high development score indicates poor socioeconomic conditions), moderate stunting decreases. However, this is accompanied by increases in severe stunting, providing that undernourishment is not too high [for the model’s equations surface plots, see Supplemental Material, Figure 2 (http://dx.doi.org/10.1289/ehp.1003311)]. As with any model, output for input variable values falling outside the range within which the model was fitted should be interpreted with caution. In the data used to parameterize the equations, the maximum value for undernourishment was 76% (Table 1), and the surface plots suggest that above this value, stunting estimates may be invalid. In our future estimates, only undernourishment in central SSA under climate change exceeded this (80% and 81%; Table 3); although these PoU estimates are only just outside the fitting range, the resulting stunting estimates should be interpreted cautiously.

The model’s equations suggest that, as either food access or general socioeconomic conditions worsen, severe stunting increases more rapidly than moderate stunting; that is, more children shift from moderate to severe stunting than shift from no/mild stunting to moderate stunting. It is likely that this behavior is partly because the model assumes that, regardless of conditions, the distribution of access to food remains constant. This assumption is a property of the FAO undernourishment model (FAO 2003) and of our development score (i.e., the Gini coefficient is assumed to remain constant at baseline levels). We believe that allowing distributions to vary should be considered in future work.

The θ parameters have negative values. This was unexpected but, when considered in the context of the full equation and in terms of observed model behavior, the model equations predicted stunting changes as expected. Thus, if either food or nonfood causes are high and those causes are then reduced, the impact on stunting is greater than if both food and nonfood causes are high and only one variable is lowered. This suggests, as expected, that to best deal with stunting it is necessary to address both food and nonfood causes.

*Dealing with uncertainty.* It is axiomatic that there are uncertainties in any risk assessment model. In this assessment, we have addressed parametric uncertainty in the stunting model through the use of Monte Carlo simulations. Structural uncertainty will be addressed in future work by exploring nonlinear interactions. It was not possible to assess the uncertainty in the upstream models (e.g., climate models, crop models, trade model) that drive our model (i.e., the input uncertainties associated with *x_ij_* and *w_ij_*) because we lacked the necessary information. Future assessments should use a wide range of climate and socioeconomic scenarios in order to capture the uncertainty of future emission pathways and the world in which the climate impacts will occur.

## Conclusions

Previous studies have shown that climate change is likely to have negative effects on future hunger and undernutrition (Nelson et al. 2009, 2010; Parry et al. 1999, 2004; Rosenzweig and Parry 1994), and our results are consistent with these. This reinforces the evidence base for action to be taken to reduce carbon emissions and the impacts of the climate change to which we are already committed. Additionally, our model suggests that to reduce and prevent future undernutrition, it is necessary to both increase food access and improve socioeconomic conditions.

Quantifying the size of the impact presents difficulties. Our work illustrates the importance of the outcome considered—for example, undernourishment versus stunting, and moderate stunting versus severe stunting. These outcomes have different implications for adaptation and decision making (e.g., whether adaptation policies should focus only on food supplies or consider water and sanitation provision) and different implications for health (e.g., severe stunting is a much greater health threat than is moderate stunting). Further, future socioeconomic conditions must be considered; this involves both developing new data sets and designing models that recognize data constraints. Above all, because none of the above issues will be easily overcome, modeling efforts should explicitly describe their assumptions and limitations.
