# Erratum: Climate Change, Crop Yields, and Undernutrition: Development of a Model to Quantify the Impact of Climate Scenarios on Child Undernutrition

**DOI:** 10.1289/ehp.123-A118

**Published:** 2015-05-01

Lloyd SJ, Kovats RS, Chalabi Z. 2011. Environ Health Perspect 119(12):1817–1823; http://dx.doi.org/10.1289/ehp.1003311Published online 15 August 2011

Table 2 originally contained two incorrect values. In column 3 (α*_k_*), –0.052 ± 0.021 has been corrected to 0.025 ± 0.013, and –0.013 ± 0.014 has been corrected to –0.052 ± 0.021. The HTML and PDF versions of the article reflect these changes. The results in the article were calculated using the correct values for the parameter estimates and remain unchanged.

The authors regret the errors.

